# Effect of Performance Feedback on Community Health Workers’ Motivation and Performance in Madhya Pradesh, India: A Randomized Controlled Trial

**DOI:** 10.2196/publichealth.3381

**Published:** 2016-12-07

**Authors:** Sangya Kaphle, Michael Matheke-Fischer, Neal Lesh

**Affiliations:** ^1^ Dimagi Software Innovations Cambridge, MA United States; ^2^ Real Medicines Foundation 11700 National Blvd, Suite 234 Los Angeles, CA United States

**Keywords:** community health workers, performance feedback, motivation, supportive supervision, mHealth apps

## Abstract

**Background:**

Small-scale community health worker (CHW) programs provide basic health services and strengthen health systems in resource-poor settings. This paper focuses on improving CHW performance by providing individual feedback to CHWs working with an mHealth program to address malnutrition in children younger than 5 years.

**Objective:**

The paper aims to evaluate the immediate and retention effects of providing performance feedback and supportive supervision on CHW motivation and performance for CHWs working with an mHealth platform to reduce malnutrition in five districts of Madhya Pradesh, India. We expected a positive impact on CHW performance for the indicator they received feedback on. Performance on indicators the CHW did not receive feedback on was not expected to change.

**Methods:**

In a randomized controlled trial, 60 CHWs were randomized into three treatment groups based on overall baseline performance ranks to achieve balanced treatment groups. Data for each treatment indicator were analyzed with the other two treatments acting as the control. In total, 10 CHWs were lost to follow-up. There were three performance indicators: case activity, form submissions, and duration of counseling. Each group received weekly calls to provide performance targets and discuss their performance on the specific indicator they were allocated to as well as any challenges or technical issues faced during the week for a 6-week period. Data were collected for a further 4 weeks to assess intertemporal sustained effects of the intervention.

**Results:**

We found positive and significant impacts on duration of counseling, whereas case activity and number of form submissions did not show significant improvements as a result of the intervention. We found a moderate to large effect (Glass’s delta=0.97, *P*=.004) of providing performance feedback on counseling times in the initial 6 weeks. These effects were sustained in the postintervention period (Glass’s delta=1.69, *P*<.001). The counseling times decreased slightly from the intervention to postintervention period by 2.14 minutes (*P*=.01). Case activity improved for all CHWs after the intervention. We also performed the analysis by replacing the CHWs lost to follow-up with those in their treatment groups with the closest ranks in baseline performance and found similar results.

**Conclusions:**

Calls providing performance feedback are effective in improving CHW motivation and performance. Providing feedback had a positive effect on performance in the case of duration of counseling. The results suggest that difficulty in achieving the performance target can affect results of performance feedback. Regardless of the performance information disclosed, calls can improve performance due to elements of supportive supervision included in the calls encouraging CHW motivation.

## Introduction

The success of small-scale community health worker (CHW) programs in providing basic health services and strengthening health systems in resource-poor settings is well documented in the literature [1-4]. CHWs operating in small-scale, well-managed projects can be effective agents of change, but often even small-scale programs lack adequate focus, reporting and documentation, training, monitoring, and supervisory and support mechanisms to encourage and motivate the CHWs to excel at their jobs [5,6].

Health worker motivation is identified as an important determinant of performance by many studies [7-10]. Other than financial incentives, important determinants of performance and motivation for CHWs include work conditions, training, audit and feedback, reminders, and supportive supervision [7,8]. Multifaceted interventions that target more than one of these factors are more likely to improve performance than single interventions [8]. Appreciation by managers, colleagues, and the community, as well as a stable job, income, and training are additional motivating factors for CHWs [9,11-13].

Supportive supervision targeting health workers’ knowledge and skills, motivation, and adherence to correct practices provide incentives that positively impact performance [8,11]. In addition to performance monitoring and providing feedback, providing health workers with specific targets can also improve CHW performance. Opening two-way communication channels reduces the sense of isolation that many CHWs face while working in remote areas and can increase internal motivation, positively impacting health worker performance and quality of care [8].

In this study, we focus on improving CHW performance by providing individual feedback on specific indicators to CHWs working in an international nongovernmental organization-run mHealth program to address malnutrition in children younger than 5 years. We open two-way communication channels by placing weekly calls to CHWs to provide feedback on their performance and discuss any challenges they are facing in their work. The aim of the intervention is to gain improvement in the performance indicators, through improvement in CHW motivation, knowledge, skills, and adherence to reporting standards. With improved ability to monitor performance and provide targeted support to CHWs, we hope to improve their motivation and performance.

### Analytical Framework

#### Intrinsic and Extrinsic Motivation

Many theories of motivation distinguish between intrinsic and extrinsic work motivation. Intrinsic motivation involves people deriving satisfaction from doing the activity because they find the activity itself rewarding [14]. On the other hand, extrinsic motivation requires “an instrumentality” between the activity and a separable consequence, such as tangible or verbal rewards [14,15].

In most work contexts, both extrinsic and intrinsic motivations come into play and affect worker performance. Although early research supported the additivity of intrinsic and extrinsic motivation, more recent work has also highlighted the tradeoffs between the two, with external rewards designed to enhance external motivation detracting from internal motivation [16,17]. Verbal rewards and feedback are found to add to intrinsic motivation compared with more tangible rewards [18-20].

#### Self-Determination Theory

The complex relationships between intrinsic and extrinsic motivation is best explained by self-determination theory (SDT), which distinguishes between autonomous motivation, analogous to intrinsic motivation, and controlled motivation, analogous to extrinsic motivation. Any behavior falls somewhere on the continuum of controlled to autonomous motivation [14]. A behavior fueled by external motivators can undergo a process of internalization, whereby it is “taken in” and becomes autonomous to the individual [21].

**Figure 1:**
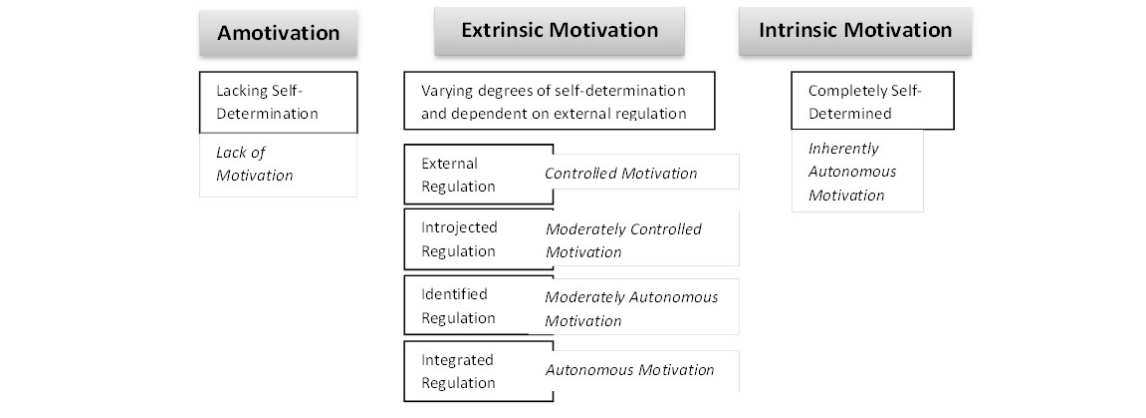
Self-determination continuum of motivation.

Figure 1 shows the internalization continuum described by SDT. Adapted from Gagne and Deci [22], Figure 1 describes the self-determination continuum of motivation from amotivation where the individual is completely lacking in motivation and there are no intentional regulations present, degrees of extrinsic motivation where contingencies and rewards are present and internalized to varying degrees resulting in degrees of self-determination, to intrinsic motivation where the individual’s motivation is completely self-determined. It shows four different levels of internalization described by the theory: (1) external regulation, in which the regulation has not been internalized and depends on contingencies of rewards or punishment; (2) introjection in which a regulation has been taken in, but has not been accepted as the person’s own and it is still controlling the person’s behavior; (3) identification, in which the regulation is more in-line with the person’s values, personal goals, and identities, and the behavior has an internal perceived locus of causality; and (4) integrated regulation in which people have the sense that the behavior is more central to their identity, it emanates from their sense of self, and is seen as self-determined and autonomous [14].

SDT postulates that increased autonomy, competence, and relatedness are the three factors or “needs” that underlie intrinsic motivation and people need to feel autonomous, competent, and need to relate to the work environment to maintain their intrinsic motivation. When all three needs are satisfied, integration is more likely to be achieved. Of these factors, autonomy support, including choice, feedback, and positive interpersonal climate with open communication and empathy, has been identified as more important for internalization [23,24]. Full internalization of external motivation has shown to yield increases in performance and work outcomes [14,25].

#### Goal-Setting Theory

Locke et al’s [26] goal-setting theory posits that goals are effective in enhancing performance when (1) they are specific, optimally difficult, and have high valence; and (2) workers understand what behavior is needed to achieve those goals and feel competent to carry out those behaviors. Goals affect performance through four mechanisms: (1) they direct effort toward goal-relevant activities and away from irrelevant activities, (2) they have an energizing function and higher goals draw greater efforts, (3) they affect persistence, and (4) they affect action indirectly by leading to discovery or use of task-relevant knowledge and skills [27].

Ensuring that the goals are viewed as important and increase self-efficacy are the two important factors that lead to goal commitment and acceptance. Goal commitment is important and the goal-performance relationship is strongest when individuals are committed to their goals. Feedback is another important factor and it is important to know one’s progress against a target to adjust the level of effort or performance strategies to match the requirements of the target. Many studies have found that a combination of goals and feedback is more effective than goals alone [28-32].

Many studies set in different countries and contexts have established that the relationship between goal setting and increased performance is reliable, although some negative effects of goal setting, such as fraudulent reporting, are possible outcomes [33].

#### Community Health Worker Motivation, Goal Setting, and Performance

Our analytical framework rests on SDT and goal-setting theory, whereby organizational goals and behavioral standards are internalized by the CHW, enhancing autonomous motivation and performance. We expect that by providing targets, performance feedback, and supportive supervision through weekly phone calls, we will influence the three factors (autonomy, competence, and relatedness) that lead to greater internalization of the external regulation and goals and this will thus increase CHW performance and it will be sustained over time.

**Figure 2:**
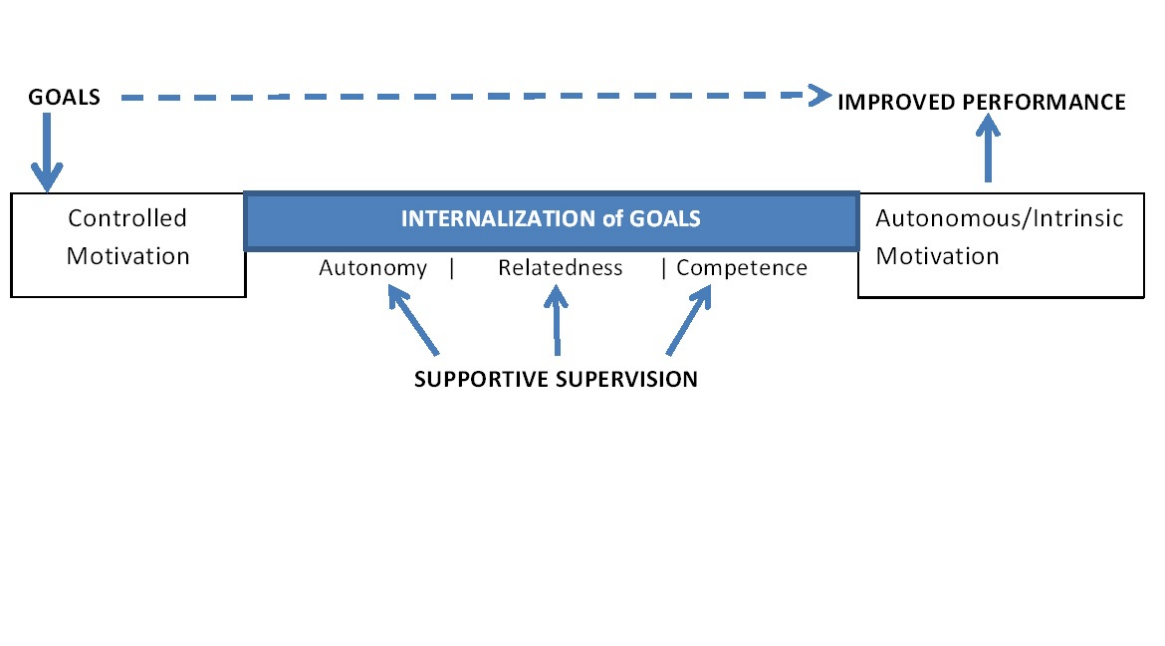
Conceptual framework: community health worker motivation, goal setting, and performance.

Figure 2 presents the conceptual framework used to develop our intervention. Since goal setting alone can lead to increased performance [26], CHWs are provided with performance targets. Supportive supervision that makes health workers feel cared for and provides recognition, appreciation, and encouragement supports autonomy in the CHW and enhances intrinsic motivation [34]. The CHWs working in remote contexts where interactions with peers and the organization is limited will have a greater sense of relatedness with regular two-way communication with someone they perceive to be higher up in the organizational structure. Setting clear goals and targets and providing performance feedback and need-based training to improve gaps in performance, improve knowledge, and set clear guidelines for practices and reporting standards can also be effective in promoting internalization and improving CHW self-efficacy, competence, and performance. A combination of goal setting and a performance feedback loop providing supportive supervision to CHWs works as a multifaceted incentive aimed at improving CHW motivation.

#### Supportive Supervision, Motivation and Performance of Community Health Workers

Regular and reliable supervision is cited as a missing link in increasing CHW performance in India [35]. Supportive supervision, community recognition and respect, peer support and learning, community information systems, and having clear roles, responsibilities, and targets are among other nonmonetary factors that incentivize CHW performance [34-41].

Appreciation and encouragement are important elements of supportive supervision. Empirical studies find positive effects of verbal rewards on intrinsic motivation and performance [42,43]. Other recent studies also identify lack of appreciation and nonrecognition of performance as demotivators for health workers [11-13]. Communicating goals clearly and making health workers feel supported and cared for are also deemed important for motivation [44].

### The Project in Madhya Pradesh, India

Real Medicine Foundation (RMF) has been running a mobile health project using CommCare, with 60 community nutrition experts, targeting infant malnutrition in 600 villages located in five districts of Madhya Pradesh, India: Jhabua, Alirajpur, Barwani, Khandwa, and Khargone. Their aim is to increase communities’ awareness about malnutrition, mobilize communities to increase demands for treatments and services to alleviate malnutrition, and strengthen the capacity of frontline workers such as Anganwadi workers in combatting malnutrition. The community nutrition experts have been working since 2010 and the program has been in effect with CommCare since July 2011.

Since the program began, RMF has successfully treated 2157 children at nutrition rehabilitation centers, improved the nutritional status of 24,822 children suffering from moderate and severe acute malnutrition, and trained more than 329,780 individuals on malnutrition prevention, awareness, and treatment. RMF works closely with the Government of Madhya Pradesh.

The community nutrition experts in the RMF program are monitored and supervised primarily via their district supervisor. Although the community nutrition experts do receive some feedback from their district supervisors, they do not receive any individual-level feedback on performance metrics. The community nutrition experts attend a meeting at the district headquarters every 15 days. The district supervisor reviews the reports the community nutrition experts submit on child health status, discusses issues that the community nutrition experts have faced in the field, and develops a roadmap for the next 15 days. There is general acknowledgment of the community nutrition experts who have performed well for that period. The district supervisor also gives specific acknowledgment to community nutrition experts when they excel in the group environment to motivate other community nutrition experts to improve their performance. The district supervisor also accompanies the community nutrition experts on home visits to monitor performance. She spends the entire day with the community nutrition expert covering the households she visits and monitors the community nutrition expert’s work closely. She provides individual feedback/support at this time, which includes supporting community nutrition experts to motivate households that are resistant to admitting their child to the nutrition rehabilitation centers and supervising reporting (ie, reviewing how the data are entered on CommCare or their paper-based forms).

#### Motivators for the Community Nutrition Experts

Focus group discussions and unstructured interviews with the community nutrition experts were conducted to gain insights on their goals, motivation, challenges, and support structures affecting their work. We conducted two focus group discussions with three and 10 community nutrition experts, respectively, and interviewed five community nutrition experts and all five district supervisors to gain better understanding of what motivates these health workers. Field observations shadowing five community nutrition experts also informed understanding of the community nutrition experts work structure and use of their mobile app.

The motivation goals of the community nutrition experts seem to be rooted in experience and the contributions they make to their households as a result of their job. Most community nutrition experts did not have prior work experience and most mentioned that they could not imagine going back to the situation when they did not work. They derive intrinsic motivation from their professional conscience and the visible improvements they see in the nutritional status of children in their catchment area. Working in the communities for 2 to 3 years, they form good relationships with the villagers. As a result, a negative outcome, such as the death of a patient, can be demotivating for them. The financial contributions they make to their households are a major motivational factor because their salary has made a significant impact on their household living standards. Their experiences have made them more confident and eager to learn more, and in many cases enlisted a desire to progress further and deal with more responsibility and gain higher salaries, although most do not have any set goals toward this objective. Very few community nutrition experts have set life or career goals for themselves. Their motivation seems to be based on a combination of intrinsic and extrinsic factors.

## Methods

In this study, we examined the impact of performance feedback on CHW performance and motivation in a randomized controlled trial with a sample of 60 CHWs who were part of the community nutrition program with RMF in Madhya Pradesh, India.

### Study Design

#### The Intervention

In their article addressing ways to improve CHW performance in India, Bajpaai and Dholakia [35] suggested additional monitoring done with minimal paperwork to improve performance of accredited social health activists. Feedback mechanisms, such as text messages or telephone complaint services, were also suggested as viable channels to provide and receive efficient feedback.

To communicate detailed performance feedback, we set up a call center, placing weekly calls to the community nutrition experts and relaying feedback on performance metrics. Phone calls provided a way to discuss and receive feedback from the community nutrition experts regarding any work-related issues, personal needs, mistraining, or technical difficulties they might have been having with the mobile app, which could be escalated to district supervisors so that they could be resolved quickly.

The European Foundation of Quality Management identifies the following human resource management principles as effective motivators for health works: supervision schemes, recognition schemes, performance management, training and professional development, leadership, participation mechanisms, and intraorganizational communication processes [44]. These motivators are effective in promoting internalization of controlled motivation by supporting perceptions of autonomy, relatedness, and competence experienced by health workers. Our intervention to provide performance feedback to community nutrition experts factored in six of these seven criteria. The community nutrition experts received supportive supervision, recognition for their work, performance feedback, training in problem areas regarding use of their app, and they had an opportunity to contribute to the program by discussing problems and challenges. Any issues identified during the calls were escalated to the program coordinator, thus contributing to intraorganization communication. Table 1 describes elements of the intervention and how they were related to supporting autonomy, competence, and relatedness to facilitate internalization and achieve sustained behavior change in the absence of the intervention.

**Table 1:** Elements of the intervention and the factors supporting internalization.

Elements of the intervention	Internalization factors	Rationale
Greetings, introductions, discuss well-being, ask if good time to call	Relatedness, autonomy	Discussing well-being of the community nutrition expert and family displays concern and empathy, and supports autonomy and relatedness because the community nutrition expert can voice any personal difficulties affecting her work.
Targets and performance feedback	Competence, autonomy	Providing performance feedback against goals targets competence and autonomy by discussing gaps in performance and proving the community nutrition expert has the means to assess her own performance as well.
Congratulations/encouragement	Autonomy	Encouragement and positive feedback increase autonomy by instilling a sense of self into the community nutrition expert’s work.
Reminders and retraining on app or work flow	Competence	Retraining and reminders on the community nutrition expert’s desired workflow or on how to use the app increases their competence.
Discuss work challenges and ways to support their work	Autonomy, relatedness	Participation and being able to discuss any challenges or issues will increase autonomy by empowering the community nutrition expert to provide input. Discussing concerns of others improves relatedness.
Technical support regarding phone or app	Relatedness, competence	Any technical problems arising on the phone can be fixed and community nutrition expert will be better able to perform the job. Discussing these issues will also improve relatedness
Reminder of target and next call, goodbyes	Autonomy, relatedness	We provide targets with the aim to improve autonomy by giving the community nutrition expert the information to do her work. It could seem like external pressure, although it is communicated positively. Warm, encouraging, interpersonal communication supports relatedness and autonomy.

#### Performance Indicators

Three performance indicators and respective targets were identified by the RMF program as relevant indicators to measure the performance of the community nutrition experts: (1) case activity, (2) number of infant and child health form submissions, and (3) duration of counseling.

##### Case Activity

Case activity was defined as the number of clients out of total clients that were visited in the last 2 weeks. The target was to visit all the registered clients every two weeks. By the end of each 2-week period, each community nutrition expert’s case activity was expected to be 100%, regardless of the number of clients registered. The case activity metric monitored the community nutrition expert’s coverage of the villages and clients and provided a measure for access to care available to the communities they served. Goal-setting theory suggests that goal difficulty has an inverse relationship with effort, with moderately difficult goals drawing the most effort out the individual and easy and very difficult goals drawing less effort and motivation to meet the target [27]. Because the community nutrition experts had different numbers of clients, the degree of difficulty of achieving this target was not consistent across the 60 community nutrition experts.

##### Form Submissions

The form submissions indicator was the total number of infant health and nutrition forms submitted in the last week. This form collects information on the child’s nutritional and health status and tracks their nutritional progress at each follow-up visit by the community nutrition expert. The target here was for each community nutrition expert to fill out this form each time she visited a household to register or follow up with a child. Hence, the number of form submissions was measured against the total number of clients visited in the last week (case activity), which provided the target for this particular indicator. The community nutrition expert could exceed the target on this metric because case activity did not register repeat visits within a period as a new visit or account for more than one form submitted during a visit. Hence, the number of form submissions for the infant health and nutrition form could be higher than the case activity if the community nutrition expert visited the same case more than once during the week. The target for form submissions was measured based on case activity; the community nutrition expert was expected to fill out at least as many infant health and nutrition forms as the number of clients visited in the last week.

##### Duration of Family Counseling

Family counseling duration was the adjusted mean time spent counseling families on infant nutrition in the last week. The target was for each community nutrition expert to spend at least 15 minutes counseling each family on the topics related to mother and infant nutrition. Tracking time taken by each community nutrition expert to counsel families on the importance of hygiene, nutrition, and admitting malnourished children to the nutrition rehabilitation centers provided a measure for the quality of care available to the communities. Because we wanted to ensure that each family received this minimum amount of counseling, all counseling times longer than 20 minutes were rounded down to 20 minutes during the data collection stage before any analysis. This was done in consultation with RMF, which had a target of 15 minutes for each counseling session. Tracking counseling times was made simple with CommCare, which records the time taken to complete each form and the community nutrition experts use a family counseling form to counsel the families.

#### Randomization and Intervention Stages

The 60 community nutrition experts were randomized into three treatment groups, one for each of the three performance indicators (case activity, form submissions, duration of counseling). The study period was 10 weeks in total and was divided into two stages: the intervention period and the postintervention period. In the intervention period, which lasted for 6 weeks, each community nutrition expert received a weekly phone call discussing their performance on a certain indicator against its target. During this period, the other two groups served as the control group. We had imbalanced randomization for the analysis (2:1) and each treatment had 20 community nutrition experts; therefore, each control had 40 community nutrition experts.

In the postintervention period, which lasted for 4 weeks, the community nutrition expert did not receive any feedback to examine whether the effects of the feedback in the intervention period were sustained or wore off over time. Because we were interested in studying dynamic, intertemporal effects of our intervention, this was a crucial time period for our study in which we could examine whether any effects on performance and reporting standards were lasting. For instance, if we found that results were positive and sustained over the two study periods, the effects of the intervention could be deemed more effective than if these effects were attributed to a novelty factor and did not carry over time without constant monitoring and feedback. We did not have a control group that did not receive any calls because the RMF program requested that all community nutrition experts receive some feedback on their performance.

Randomly allotting the community nutrition experts into the three treatment groups could result in lopsided treatment groups based on baseline performance. To create balanced treatment groups, we first ranked the community nutrition experts baseline performance on each of the three indicators. We gave a percentage score for each indicator by comparing the community nutrition expert’s performance on each indicator against the target for that indicator and converted these into *z* scores so that they were normalized. We then tallied up baseline performance scores for the three indicators using the *z* scores for each community nutrition expert and used this score to rank the community nutrition expert’s baseline performance. For randomization, the community nutrition experts were sorted by baseline rank in groups of three. To generate balanced treatment groups in terms of baseline performance, we randomly allocated each community nutrition expert in the group to the three treatment groups.

### Data

Data for the study on the three indicators were obtained directly from CommCareHQ, CommCare’s cloud-based server where data recorded by the community nutrition expert were stored in real time. Additional data on the community nutrition expert’s demographic characteristics, personal traits, and motivation were collected in a survey conducted at the end of the postintervention period. We collected additional qualitative data from interviews with five community nutrition experts and the district coordinators as well as a focus group discussion with 10 community nutrition experts exploring motivations and challenges faced by community nutrition experts in their work and communities at the start of the study. We also collected some qualitative information at the end of the study to follow up on what was driving the results. This was done by randomly selecting three community nutrition experts from each treatment group for follow-up interviews to assess the main drivers of any changes in performance from their perspective.

We had panel data spanning 10 weeks, in which we collected performance data on each indicator for the three treatment groups. Our dataset had some important limitations, the more pressing of which was measurement error due to technical errors arising in the app. The community nutrition experts were working in areas with low network connectivity and were using an older version of the app, which had some technical errors that were not resolved before the start of our intervention. Working in low network connectivity means that the telecommunications network required to send the collected data to the central server was often inadequate or missing to send the information; therefore, there was measurement error in the data we could access from the server. Other technical errors, such as app error or missing multimedia and other bugs, meant that sometimes community nutrition experts were unable to access the app to record and send the data to the server. Many community nutrition experts also had repeat registrations for the same client with up to five repeat entries. This also affected our data quality for the first indicator—case activity—because this was measured against the total number of registered clients. During the intervention, community nutrition experts were advised not to fill out repeat registrations as they were doing before the intervention, meaning that the number of form submissions could decrease following the intervention. Similarly, we did not have data for total clients visited for the postintervention period, meaning that we did not have the target against which to measure form submissions data for that period. Therefore, form submissions is divided into two indicators: (1) form submissions number (ie, number of forms submitted in the last week), which was analyzed in the intervention and postintervention period; and (2) form submissions proportion, the number of forms as a proportion of total clients visited in the last week, which was analyzed only in the intervention period.

#### Qualitative Data

We administered an end-line survey to all community nutrition experts who were not lost to follow-up to capture their perception of the calls. We also randomly selected three community nutrition experts from each of the three treatment groups and conducted unstructured interviews on the phone to understand what was driving the results. The interviews were conducted by the program coordinator for RMF’s nutrition program.

We also conducted two focus group discussions during the intervention with three and 10 community nutrition experts, respectively, and interviewed five community nutrition experts and all five district supervisors to gain a better understanding of what motivated these health workers. Field observations shadowing five community nutrition experts also informed understanding of the community nutrition experts work structure and use of the mobile app. No feedback calls were placed during this time to any of the community nutrition experts.

Our aim was to study the effects of providing performance feedback on CHW motivation and performance. Because motivation is difficult to measure directly, we focused on assessing the effects of the feedback calls on CHW performance on specific indicators. The effects of other motivators could not be analyzed directly because of the study design, which did not include a control group that did not receive any calls. Therefore, the main focus of the analysis and empirical strategy is on the effects of the performance feedback.

The indicators used in this study were identified as direct measures of quality and access to health care because they were important for the RMF program and for other programs using mHealth. Adherence to correct practices in reporting and utilization of the mHealth platform is important and cannot be separately measured from the performance indicators discussed subsequently because all data were collected using CommCareHQ, which does not distinguish between reporting and performance. Accurate reporting is a vital element of the community nutrition expert’s performance and we viewed any change in performance indicators due to adherence to reporting standards as an improvement in CHWs overall performance as well.

#### Feedback Procedure

The call center agent for our intervention was an anonymous call center operator, who was introduced to the community nutrition experts as a member of CommCare by RMF before the start of the intervention. The community nutrition experts were told to expect calls from CommCare and respond to the call by the RMF program. Due to unforeseen personal reasons, the call center operator changed after 3 weeks of the intervention and the researcher (SK), also introduced as a member of CommCare, placed the remainder of the calls. The community nutrition experts had no prior relationship to the call center operator or the researcher before the study. All the community nutrition experts received face-to-face interaction at the end of the study period for a survey and 18 of 60 community nutrition experts received additional visits, 13 in the form of a focus group discussion and five in the form of shadow visits to their clients during the intervention.

We began each call by greeting the community nutrition expert and asking her about her family and well-being; we then asked if she had some time to discuss her performance for that week. The community nutrition experts then received their performance feedback. The feedback was nuanced depending on whether she (1) met/exceeded her target, (2) improved a lot or little, or (3) showed no improvement in her work. All feedback included a reminder of the target, appreciation for their efforts, and a discussion of problems and solutions regarding their work. Those with little or no improvement were encouraged to meet their target and retrained on using the app and the desired workflow regarding the relevant indicator. Next, technical problems in the app were discussed and recorded to be escalated to the project coordinator. Finally, community nutrition experts received another reminder about their targets, the day and time of the next phone call, and a warm goodbye.

The mean duration of the calls was approximately 5 minutes, although the initial calls were longer. Each community nutrition expert received a call on a particular day of the week at a particular time determined by the community nutrition expert. Because the community nutrition experts lived in areas with poor network connectivity and could be busy with other work, we attempted to call every community nutrition expert five times in case she could not respond or the call did not go through. Three of these attempts were made on the day the community nutrition expert was scheduled to receive the call, one was made the following day, whereas those who were not reached during the week received an additional attempt at the end of the week when they were not at work.

### Empirical Strategy

We estimated main treatment effects and heterogeneous effects of call intensity (number of calls received) for each of the three performance indicators using a random effects model. We first estimated double difference estimates for main effects and heterogeneous effects in the intervention period, and then expanded the analysis to the postintervention period to estimate sustained effects of the intervention. The equations used to look for the main effects of the treatment for each indicator (double difference estimator, cross-partial effects, and comparing all three indicators head-to-head) as well as the equation used to estimate heterogeneous effects are presented in Multimedia Appendix 1.

### Statistical Tests

We used a range of statistical tests fitting the nature of the variables. Community nutrition expert characteristics were compared using one-way ANOVA because we were comparing the means of three different treatment groups, whereas the Fisher exact test was used for the categorical variables because of the small number of observations in some of the categories. Binomial tests were used for the binary variable “other work.” Because the performance indicators did not follow a normal distribution, following Gneezy and Rustichini [45], we used the Mann-Whitney *U* test to compare the medians of the three treatment groups to one another. We also used unpaired *t* tests with unequal variance to compare means of the treatment group with the relevant control group, and the Fisher exact test for categorical variables.

### Ethical Considerations

We took all measures possible to ensure that our study followed research governance and ethical protocols necessary for such research. The study benefited the participating community nutrition experts and the larger community where they worked by exploring ways to improve the quality and experience of the health services they provided to their communities. The study posed minimal risk to the participants and was part of the normal operational experimentation done with CommCare users. The community nutrition experts often received feedback through their district supervisors, which is in-line with the feedback provided in the intervention. There was no consequence to the community nutrition expert’s job as a result of the experiment because we did not systematically share performance data with RMF program managers making hiring and firing decisions.

## Results

**Figure 3:**
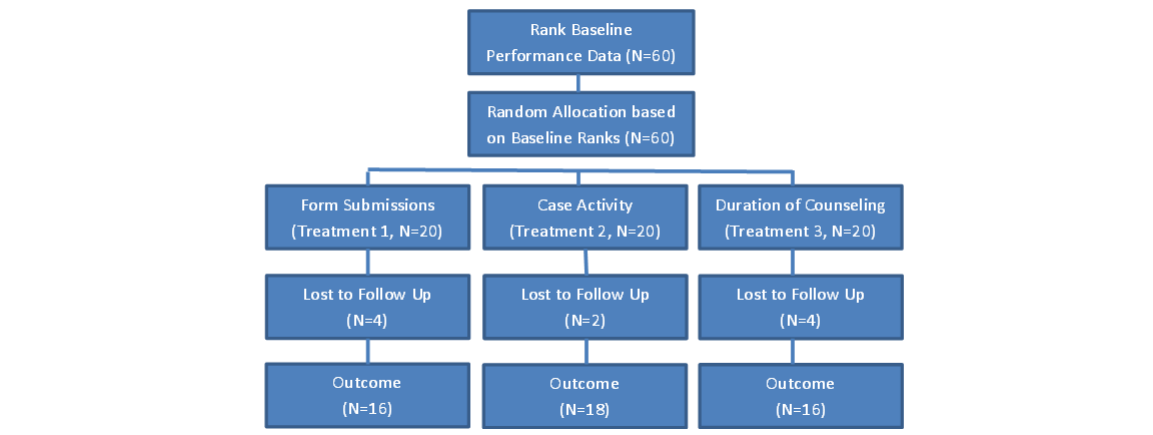
Randomization and treatment allocation of participants.

In total, 60 community nutrition experts were randomized into three treatment groups based on baseline performance. Figure 3 shows the flow of participants through the study.

### Descriptive Statistics and Baseline Comparisons

Table 2 presents summary statistics of some community nutrition expert characteristics and baseline performance indicators of the entire sample and by treatment groups.

**Table 2:** Descriptive statistics, community nutrition expert characteristics, and performance indicators.

Characteristics and indicators		Total (N=60)	Treatment group		
			Form submission (n=20)	Case activity (n=20)	Duration of counseling (n=20)
**Community nutrition expert characteristics, mean (SD)**						
	Baseline rank	30.4 (17.22)	29.95 (17.61)	30.6 (17.29)	30.65 (18.03)
	Age (years)	32.15 (7.49)	31.21 (5.84)	32.15 (6.81)	33.05 (9.54)
	Number of children in household	1.73 (1.14)	2.05 (1.02)	1.85 (1.18)	1.3 (1.13)
	Number of adults in household	3.63 (1.98)	3.42 (1.92)	3.35 (2.03)	4.1 (1.99)
	Education (years)	11.55 (2.59)	10.57 (2.24)	11.8 (2.59)	12.25 (2.73)
**Performance indicators,^a^ mean (SD)**						
	Form submissions (n)	43.68 (48.39)	41.75 (47.69)	42.4 (50.63)	46.9 (49.15)
	Form submissions (proportion)	0.42 (0.43)	0.41 (0.45)	0.37 (0.37)	0.49 (0.48)
	Case activity	17.65 (24.55)	18.33 (27.00)	16.4 (22.99)	18.20 (24.71)
	Duration of counseling (mins)	2.01 (3.24)	2.44 (4.31)	1.92 (2.69)	1.67 (2.57)

^a^ Form submissions number was measured as the number of form submissions in a week. Form submissions proportion was measured as form submissions as a proportion of total clients. Case activity was measured as percentage of total clients visited in a 2-week period. Duration of counseling was measured as duration of family counseling in minutes.

The mean age of the community nutrition experts was 32 (SD 7.49) years with mean 11.5 (SD 2.59) years of education. Approximately 24% (14/60) of the community nutrition experts were engaged in other work, including agricultural work, in addition to their job with RMF. Baseline performance indicator means for the community nutrition experts were (1) form submissions (mean 43.68, SD 48.39), (2) case activity (mean 17.65, SD 24.55), and (3) duration of counseling (mean 2.01, SD 3.24). We did not find any significant differences between the three treatment groups in terms of rank, age, education, number of children, number of adults, marital status, and year started as community nutrition expert. Similarly, baseline performance indicators also did not exhibit a difference in medians for the three treatment groups.

The treatment groups and their relevant control group were also balanced in terms of community nutrition expert characteristics and performance indicators. However, we did see some significant differences between treatment and control means for duration of counseling in terms of number of children (*P*=.04) and education (Fisher exact=0.055).

### Differential Attrition

We tested for differential attrition across the entire sample and within the three treatment groups. The attrition rate for the entire sample was high at 17% (10/60) and 10 of 60 community nutrition experts were lost to follow-up during the course of the study. Most of these were due changes in the community nutrition expert appointments made by the RMF program during the course of the study. We had differential attrition based on rank in our data because (1) the program replaced some lower-performing community nutrition experts during the course of the study (this was done outside of our intervention because the performance feedback was not shared with RMF program managers) and (2) the data in CommCareHQ was not updated at the start of the study, so some community nutrition experts who dropped out or were replaced before the start of the study were included in the baseline data, whereas their replacements were included in the end-line data (ie, we did not have baseline data for the replacements). We found differential attrition for baseline rank (*P*<.001) with lower-ranked community nutrition experts dropping out more than those with a higher ranking in terms of baseline performance indicators. We also found differential attrition significant for age (*P*=.009), with younger community nutrition experts dropping out, and year started as a community nutrition expert (*P*=.003).

We found differential attrition in terms of rank for all three treatment groups and in terms of age (*P*=.02) and year started as community nutrition expert (*P*=.04) only for those in in the duration of counseling treatment group. In terms of rank, we found significant differential attrition for duration of counseling (*P*=.01). The form submission group (*P*=.09) and the case activity group (*P*=.09) did not have significant differential attrition for rank. There was no differential attrition effect for education, number of children, and number of adults, marital status, or other work for all three intervention groups.

Lower-performing community nutrition experts in the baseline period who dropped out of the study in all three treatment groups could have affected our results.

To ensure that results were not driven as a result of the differential attrition, we re-estimated equations 1 and 4 in Multimedia Appendix 1 to account for attrition biases by matching the community nutrition experts lost to follow-up with those with the closest rank in the same treatment group. Multimedia Appendix 2 presents the results of the matching and shows the rank, age, education, marital status, number of adults, number of children, start year, and other work data for the community nutrition experts lost to follow-up and their replacements.

### Main Effects

Tables 3 and 4 present the main treatment effects from equation 1 (Multimedia Appendix 1) in the intervention period and the postintervention period to identify intertemporal, sustained effects. There are three different regression specifications for each of the three indicators. The variables were defined so that case activity, form submissions, or duration of counseling indicated the treatment group, with a value of 1 if the community nutrition expert belonged to that treatment group and 0 otherwise. The variable “after” indicated the time variable with a value of 0 if the data were from the baseline period and 1 if from the intervention or postintervention period. The variable “treatment×after” indicated our double difference estimator and was an interaction term between the treatment and time variables. Form submissions were analyzed in terms of absolute number of form submissions and proportion of form submissions to total clients visited in a 7-day period. The results to correct for differential attrition were also included in Tables 3 and 4, and show the double difference estimates after matching and replacing community nutrition experts lost to follow-up with those with the closest baseline rank in the same treatment group. The estimated coefficients give percentage point changes for case activity, form submissions proportions and absolute changes (ie, change in absolute number of form submissions), and absolute number of minutes for duration of counseling.

**Table 3:** Difference in differences (DID) estimates: impact of calls on performance indicators during the intervention stage.

Dependent variable		Case activity			Form submissions (number)			Form submissions (proportion)			Duration of counseling		
		DID	*t* (*df*)	*P*	DID	*t* (*df*)	*P*	DID	*t* (*df*)	*P*	DID	t (*df*)	*P*
**Original sample (n=300)**													
	Treatment	–3.98	–0.59 (299)	.55	–7.09	–0.97 (299)	.33	–0.042	–0.30 (299)	.76	–0.54	–0.37 (299)	.71
	After	9.27	2.65 (299)	.008	–36.94	–8.93 (299)	.001	0.22	3.31 (299)	.001	–0.09	–0.11 (299)	.91
	Treatment×after	4.66	0.80 (299)	.42	2.89	0.40 (299)	.68	–0.002	–0.02 (299)	.98	3.86	2.91 (299)	.004
	Constant	22.20	5.46 (299)	<.001	52.15	12.57 (299)	.001	0.49	6.27 (299)	.001	2.43	2.97 (299)	.003
**Correcting for attrition (n=360)**													
	Treatment	–6.57	–1.48 (359)	.13	–1.26	–0.31 (359)	.76	–0.12	–1.35 (359)	.17	–0.64	–0.49 (359)	.62
	After	12.43	4.41 (359)	<.001	–29.50	–8.80 (359)	.001	0.26	4.44 (359)	.001	0.07	0.1 (359)	.92
	Treatment×after	4.78	1.23 (359)	.21	–1.57	–0.40 (359)	.68	0.04	0.49 (359)	.62	3.48	2.88 (359)	.004
	Constant	21.78	5.28 (359)	.001	44.58	10.97 (359)	.001	0.51	5.91 (359)	.001	2.31	3.06 (359)	.002

The results showed a significant increase in performance for duration of counseling in the intervention period, whereas there were no significant treatment effects for case activity and form submissions. The interaction of counseling×after, which captured the treatment effect for receiving feedback on the duration of counseling, was significant at the 90% confidence interval, and receiving performance feedback on the duration of counseling increased mean counseling times by 3.860 minutes (*P*=.004), which had an effect size of Glass’s delta=0.974 (control group SD 2.910) and Cohen’s *d*=0.778 (pooled SD 4.976), both of which indicate a moderate to large effect of receiving duration of counseling feedback. After correcting for attrition bias, the effect size was Cohen’s *d*=0.710 (pooled SD 4.908) and Glass’s delta=0.883 (control SD 3.945).

In Table 4, analyzing the intervention and postintervention period data together, we found that the impact of receiving performance feedback on duration of counseling on mean counseling times was sustained. Counseling×after, which captured the treatment effect for receiving performance feedback on the duration of counseling during the intervention and postintervention periods, was significant and counseling times increased by a mean 4.469 minutes (*P*=.004) when we included the postintervention data, suggesting that the effects of the intervention were sustained postintervention when the community nutrition experts were not receiving feedback on their performance. This is an effect size of Glass’s delta=1.690 (control group SD of duration of counseling for the period is 3.66), and an effect size of using Cohen’s *d=* 0.813 (pooled SD of duration of counseling for the period is 5.87), both of which indicated a moderate to large effect of receiving duration of counseling. The results held after correcting for differential attrition, although we had a smaller effect size of using Glass’s delta=0.926 (control group SD of duration of counseling for the period is 4.683) and Cohen’s *d*=0.748 (pooled SD of duration of counseling for the period is 5.795). Because the results after correcting for the attrition bias were similar to the results generated by the original sample, we proceeded with the original sample for further analysis.

**Table 4:** Difference in differences (DID) estimates: impact of calls on performance indicators in the intervention and postintervention stages.

Dependent variable		Case activity			Form submissions (number)			Duration of counseling		
		DID	*t* (*df*)	*P*	DID	*t* (*df*)	*P*	DID	*t* (*df*)	*P*
**Original sample (n=500)**										
	Treatment	–3.98	–0.57 (499)	.57	–7.09	–0.95 (499)	.34	–0.54	–0.32 (499)	.75
	After	9.07	2.65 (499)	.008	–32.32	8.20 (499)	.001	0.57	0.77 (499)	.44
	Treatment×after	5.58	0.98 (499)	.33	2.032	0.29 (499)	.77	4.78	3.66 (499)	.001
	Constant	22.20	5.34 (499)	.001	52.15	12.31 (499)	.001	2.43	2.54 (499)	.01
**Correcting for attrition (n=600)**										
	Treatment	–6.06	–1.40 (599)	.16	–1.142	–0.29 (599)	.77	–0.64	–0.42 (599)	.67
	After	12.71	4.52 (599)	.001	–25.36	–7.77 (599)	.001	0.746	1.09 (599)	.27
	Treatment×after	4.45	1.2 (599)	.023	–1.39	–0.37 (599)	.71	4.34	3.65 (599)	.001
	Constant	21.43	5.18 (599)	.001	44.51	10.7 (599)	.001	2.31	2.62 (599)	.009

We tested for statistical significance of the marginal difference in duration of counseling in the intervention and postintervention periods by restricting the data to only include observations in the postintervention period (weeks 7-10). We ran two specifications, first to look for changes in duration of counseling from the baseline period of week 1 and second where we set the baseline at the end of the intervention period in week 6. At week 1, duration of counseling was mean 2.45 (SD 3.80) minutes for the treatment group (n=34) and mean 1.89 (SD 2.83) minutes for the control group (n=16). At week 6, duration of counseling was mean 3.15 (SD 3.63) minutes for the control group (n=16) and mean 10.70 (SD 6.50) minutes for the treatment group (n=34). At weeks 7 to 10, duration of counseling was mean 3.82 (SD 5.53) minutes for the control group (n=64) and mean 9.22 (SD 7.71) minutes for the treatment group (n=136).

**Figure 4:**
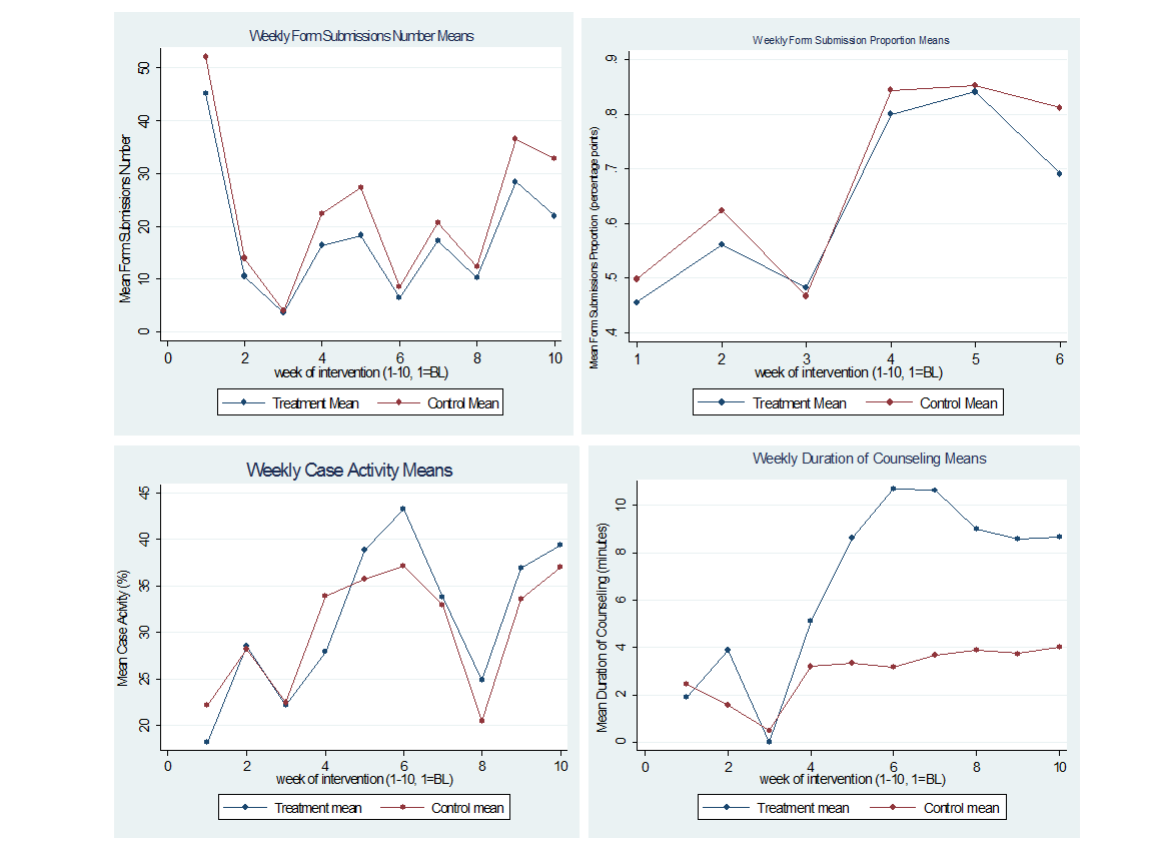
Treatment and control means in intervention and postintervention stages. Duration of counseling means are the mean duration of counseling per form submitted. BL: baseline.

The results of the two specifications are presented in Table 5. We found that there was positive and significant impact of the calls on duration of counseling performance from baseline to the postintervention period using only postintervention data (coefficient=5.939, *P*<.001). Setting week 6 as the baseline and comparing counseling times at the end of the intervention period to the postintervention period, we found that counseling times fell significantly (coefficient=–2.14, *P*=.02). This result is highlighted in Figure 4, which shows weekly mean counseling times for treatment and control groups over both study periods. This result shows that performance feedback is an important driver for performance where duration of counseling is concerned. Although we found positive intertemporal effects and receiving performance feedback led to large sustained improvements in counseling times for community nutrition experts, they dropped off in the postintervention period when they were not receiving feedback. The community nutrition experts were retrained on using the counseling forms and were reminded of their targets during the calls, the effects of which were sustained through to the postintervention period. However, they performed better while receiving performance feedback and reminders of their targets, and the feedback seemed to have intensified their internal motivation to perform and meet their goals.

**Table 5:** Impact of performance feedback on duration of counseling in the postintervention period looking for sustained effects in performance.

Dependent variable	Duration of counseling					
	Baseline=week 1 (n=250)			Baseline=week 6 (n=250)		
	Coefficient	*t* (249)	*P*	Coefficient	*t* (249)	*P*
Duration of counseling	–0.536	–0.30	.76	7.552	4.09	.001
After (baseline=week 1)	1.390	2.07	.39			
Counseling×after (baseline=week 1)	5.939	5.00	.001			
After (baseline=week 6)				0.672	1.34	.18
Counseling×after (baseline=week 6)				–2.149	–2.43	.02
Constant	2.433	2.4	.02	3.151	3.02	.002

There were no significant effects of receiving feedback on form submissions or case activity, in the intervention and postintervention periods. In the postintervention period, we did not have data for the total number of clients visited in the 7-day period; hence, we could not include form submissions as proportion of clients visited as a variable. Before the intervention, both form submissions and case activity were lower in the treatment group; however, this difference was not significant. There seemed to be an overall decrease in form submissions for all community nutrition experts (after) and the pre-post changes in the treatment and control groups were essentially the same. This could be explained by the fact that most community nutrition experts had multiple registrations of the same case in their apps, with some community nutrition experts having up to five duplicate registrations of the same case. Before the intervention, the community nutrition experts submitted up to five different infant and nutrition forms for each of the duplicate registrations. This meant that baseline form submissions were much higher than appropriate because the community nutrition experts submitted many duplicate forms for the same case. The performance feedback and retraining given to all community nutrition experts during the study period by the program reinforced that there was no need to submit duplicate forms despite the duplicate registrations, and only one infant health and nutrition form should be submitted per visit rather than submitting multiple forms for each of the duplicate registrations. This explains the overall decrease in form submissions in the intervention and postintervention period. Our interpretation was supported by the increase in form submissions proportional to number of clients visited in specification 3 for the control group and a slightly lower effect for the treatment group in the postintervention period.

Although case activity also showed an increase in performance in both the intervention and postintervention periods, the results were not significant. However, there was an overall increase in case activity for community nutrition experts in the control group in the intervention and postintervention period. The interpretation here could be that regardless of the information received during the calls, receiving calls boosted the community nutrition experts’ case activity. However, because we did not have a control group that did not receive calls, we could not estimate the effect of calling itself. The results are confounded by other factors, which could have caused a secular trend of increasing performance on case activity, including retraining during the intervention period by RMF, which alone could explain the increased performance after the intervention, independent of the intervention itself.

Although there were some program-wide changes to increase community nutrition expert performance during our intervention confounding the effects, including distribution of an additional paper-based job aid for counseling and retraining on CommCare for all the community nutrition experts, our intervention played an important part in increasing community nutrition expert performance for case activity. One interpretation of these results could be that regardless of the information disclosed in the calls, receiving calls boosted community nutrition expert performance by increasing internal motivation and increasing interest in their work. Although providing performance feedback against a target was an important element of our intervention, the feedback calls went beyond providing simple feedback and fluid discussions about the community nutrition experts’ technical and program-related challenges, and some personal/family matters were an important part of the calls. Our intervention strengthened supervisory structures already present in the program, regularly communicating the technical and program-related challenges to the project coordinator and the community nutrition experts’ immediate supervisors, who were able to resolve these more quickly, contributing to the increased case activity for all community nutrition experts in the postintervention stages. Additionally, the calls also resolved problems the community nutrition experts were having in using their app, which could also have contributed to the increasing their case activity because all data was collected through their CommCare apps.

The challenge in drawing out the impact of the calls is that we did not have a control group that did not receive any calls in our design. However, our interpretation is supported by the qualitative work supporting this study where community nutrition experts mentioned that the calls were an important factor in motivating and engaging them, increasing their interest in their work. The results presented in Table 3 show that the calls were seen as an indication that the program and CommCare were taking an interest in their work and the community nutrition experts mentioned that it increased their job satisfaction and motivation as a result. In focus group discussions, community nutrition experts mentioned that their interest and motivation grew when they engaged with something new on a periodic basis and had the opportunity to interact with people who were not a part of their usual day-to-day interactions. The calls were able to provide them with such a service, targeting autonomy, relatedness, and competence, and contributing to strengthening their intrinsic motivation and interest toward their work.

The end-line survey administered to all community nutrition experts suggested that community nutrition experts perceived the calls as a motivator having a positive effect on their performance. In all, 86% (38/44) reported that they felt the calls improved their performance, 79% (34/43) felt the calls motivated them a lot to do their work, and 45% (20/44) found performance feedback to be the most effective component of the calls for motivation. In addition, 75% (33/44) reported that their technical problems were resolved faster as a result of the calls and 100% (44/44) of community nutrition experts felt that their knowledge about using CommCare had improved as a result of the calls and 94% (43/46) would sign up for this service. These results support our interpretation that the calls improved community nutrition experts’ intrinsic motivation by supporting competence, relatedness, and autonomy, leading to improvements in case activity for all community nutrition experts.

### Cross-Partial Effects

To determine whether receiving feedback on any indicator affected case activity performance or performance on the remaining two indicators, we estimated cross-partial effects of providing performance feedback on one indicator on the performance on the other two indicators. For each of the treatments, the remaining two groups acted as a pooled control and we were interested in estimating cross-partial effects of receiving feedback on case activity, on performance in duration of counseling, or form submissions. We present the analysis for the postintervention period because this generated higher power and was a more policy-relevant result. However, our analysis showed the results in the intervention period were similar to those in the postintervention period. There was one cross-partial effect where feedback on one indicator affected performance on another indicator. It negatively affected duration of counseling by approximately 3.39 minutes (*P*=.01), suggesting that receiving duration of counseling feedback was more effective in increasing counseling times than receiving form submissions feedback. We did not find any cross-partial effects for the remaining two indicators.

### Comparing the Three Groups Head-to-Head

We compared each of the three treatment groups head-to-head and estimated how feedback on each indicator affected each treatment group separately. The reference group in each specification was the treatment group. The results were in-line with our analysis from equation 1 (Multimedia Appendix 1); there were significant improvements in counseling times with mean counseling times of community nutrition experts in the duration of counseling group increasing compared with those in the form submissions or case activity treatment groups. Receiving feedback on form submissions or case activity did not significantly impact performance on that indicator compared to the two other groups. The results support the previous findings, with no baseline differences between the three treatment groups, and duration of counseling feedback affecting counseling times differently than case activity or form submissions feedback (Table 6).

**Table 6:** Head-to-head comparison of treatment groups (n=500).

Variable		Case activity			Form submissions (number)			Duration of counseling		
		Coefficient	*t* (499)	*P*	Coefficient	*t* (499)	*P*	Coefficient	*t* (499)	*P*
**Treatment**										
	Case activity	—	—	—	1.99	0.23	.81	0.19	0.09	.92
	Form submissions	3.66	0.46	.64	—	—	—	0.94	0.47	.63
	Duration of counseling	4.29	0.54	.58	12.81	1.46	.14	—	—	—
After		4.52	0.93	.35	–3.29	–5.28	.001	5.35	4.97	.001
**Treatment×after**										
	Case activity	10.13	1.53	.12	2.36	0.3	.76	–4.09	–2.77	.005
	Duration of counseling	9.10	1.33	.18	–6.97	–0.86	.39			
	Form submission							–5.56	–3.65	.001
Constant		18.23	3.31		45.06	7.28		1.89	1.35	.17

### Heterogeneous Effects

The distribution of calls received among the 50 community nutrition experts who were included in the analysis was as follows: 10% (5/50) of community nutrition experts received zero calls, 6% (3/50) received one call, 6% (3/50) received two calls, 16% (8/50) received three calls, 14% (7/50) received four calls, 10% (5/50) received five calls, and 38% (19/50) received all six calls.

We wanted to examine whether the effect of our intervention varied across the intensity of treatment (estimate equation 4 in Multimedia Appendix 1). We interacted the number of calls received with the treatment and time period (intervention and postintervention) to look for heterogeneous impacts based on intensity of exposure to treatment. Table 7 presents the heterogeneous impacts of call intensity for the postintervention period. The estimates indicated that call intensity mattered for sustaining increases in duration of counseling and counseling times increased by approximately 1.366 minutes (*P*=.04) with each additional call in the postintervention period. The estimates did not indicate any heterogeneous impacts of call intensity on the other two indicators in the intervention or the postintervention periods. The results held after accounting for differential attrition.

**Table 7:** Heterogeneous effects: the effect of receiving more calls on performance.

Variable		Case activity			Form submissions, number			Duration of counseling		
		Coefficient	*t* (*df*)	*P*	Coefficient	*t* (*df*)	*P*	Coefficient	*t* (*df*)	*P*
**Original sample (n=500)**										
	Treatment	–4.70	0.28 (499)	.77	8.05	0.52 (499)	.60	–1.45	–0.40 (499)	.69
	After	22.07	3.15 (499)	.001	0.21	0.02 (499)	.98	–0.72	–0.45 (499)	.65
	Calls	4.52	2.26 (499)	.02	7.98	3.70 (499)	.001	–0.39	–0.89 (499)	.37
	Treatment×after	13.98	1.03 (499)	.30	–9.97	–0.70 (499)	.48	–0.45	–0.16 (499)	.87
	Treatment×calls	–0.54	–0.15 (499)	.88	–2.96	–0.84 (499)	.40	0.22	0.27 (499)	.79
	After×calls	–3.47	–2.12 (499)	.03	–7.79	–3.93 (499)	.001	0.32	0.90 (499)	.37
	Treatment×after×calls	–1.35	–0.47 (499)	.63	2.12	0.65 (499)	.51	1.37	2.11 (499)	.04
	Constant	5.24	0.61 (499)	.54	18.81	1.89 (499)	.06	4.05	1.99 (499)	.05
**Correcting for attrition (n=600)**										
	Treatment	–6.28	–1.35 (599)	.17	–0.39	–0.10 (599)	.92	–1.18	–0.38 (599)	.70
	After	26.17	4.72 (599)	.001	4.31	0.64 (599)	.52	–0.15	–0.11 (599)	.91
	Calls	4.28	2.75 (599)	.006	8.04	5.08 (599)	.001	–0.26	–0.65 (599)	.52
	Treatment×after	10.86	1.40 (599)	.16	–0.77	–0.11 (599)	.91	–0.90	–0.36 (599)	.72
	Treatment×calls	–0.18	–0.12 (599)	.90	–0.64	–0.39 (599)	.69	0.14	0.19 (599)	.84
	After×calls	–3.67	–2.67 (599)	.007	–7.93	–4.97 (599)	.001	0.24	0.75 (599)	.45
	Treatment×after×calls	–1.26	–0.64 (599)	.52	–0.02	–0.01 (599)	.99	1.42	2.43 (599)	.02
	Constant	5.71	0.83 (599)	.40	14.44	2.06 (599)	.39	3.28	1.91 (599)	.06

The number of calls received per community nutrition expert suggested that the number of calls received, regardless of the indicator for feedback, had a positive impact in pre-post differences in the control group. Because all community nutrition experts received calls, this finding suggested that regardless of the information disclosed in the calls, receiving calls improved performance by providing supportive supervision and increasing intrinsic motivation by including appreciation, encouragement, avenues to discuss problems and solutions, and solving technical difficulties.

## Discussion

Our findings show some interesting differences in the effect our intervention has on the different performance indicators. We found positive impacts on duration of counseling sustained in the postintervention period, whereas case activity and form submissions showed no significant effects. We also found heterogeneous impacts of call intensity for duration of counseling, with each additional call improving the community nutrition expert’s counseling times further. There was an overall increase in case activity for all community nutrition experts, whereas the number of form submissions decreased due to multiple registrations and duplicate form submissions declining indicating improvement in overall reporting standards. We discuss possible reasons for these differing results subsequently, further supplemented with qualitative interviews with community nutrition experts to better understand the results.

First, goal-setting theory suggests that the difficulty of the target matters for motivation and performance, and moderately difficult targets induce more effort and motivation compared to difficult or easier targets [27]. In our study, targets for duration of counseling were easier to achieve because they could be achieved by spending more time with the clients that the community nutrition expert was visiting. For case activity, the target was much more difficult, especially if the community nutrition expert had a lot of cases that she had to follow up on. The target for form submissions was related to case activity and should be an easy one. However, we saw a sharp decline in form submissions for all community nutrition experts because they were asked to stop submitting duplicate registrations and infant health and nutrition forms for a single client.

Second, the data quality for duration of counseling is likely to include less measurement error compared with data capturing case activity and form submissions. The community nutrition experts had many duplicate registrations in their app for each client due to mistraining issues at the beginning of the CommCare program. As a result, some community nutrition experts had up to 400 or 500 clients registered in their app with most of these being duplicate registrations. Case activity was measured against total clients and community nutrition experts were regularly submitting multiple forms (for each of the duplicate registrations) skewing form submissions and case activity data. The overall decrease in form submissions number in both the treatment and control groups can be explained by the retraining from RMF, which reinforced that the community nutrition experts should not submit multiple forms for the same visit in spite of the duplicate registrations. Our intervention also included training on how to correctly use the app and reinforced the appropriate workflow that the community nutrition experts should adopt, which also contributed to the reduction in the number of form submissions. Form submission as a proportion of clients visited did not show any significant decrease supporting our reasoning.

Third, case activity was affected by the holiday period of Diwali, which fell around week 3 of the intervention period. Figure 4 shows the weekly means for case activity for the treatment and control groups, both of which were close to zero during the holiday period. Duration of counseling was not affected by the holiday period because if the community nutrition expert did not visit and counsel a family, it did not affect the mean counseling time.

Fourth, all community nutrition experts also received a paper-based counseling aid during the study, which could also drive the increase in duration of counseling. However, we expect this aid to affect counseling times for all community nutrition experts and not just those in the treatment group. It could also be the case that the additional counseling aid interacted with receiving duration of counseling feedback by positively affecting duration of counseling and, in the absence of the additional counseling aid, there would be no significant effects on duration of counseling. However, we cannot perform this analysis due to data constraints.

Lastly, the significance of differential attrition by rank is higher in the duration of counseling treatment group than in the other two groups. Thus, it could be the case that the duration of counseling results are being driven by differential attrition, although this is unlikely because all groups show significant differential attrition by rank.

We expect that the changes in performance also include improvements in reporting standards and are being driven by increased effort on the part of the community nutrition expert, where our intervention improved community nutrition expert motivation and encouraged and increased her interest toward her work. External motivation played a part where community nutrition experts are weary of the consequences of bad performance, such as getting fired or reprimanded by the program. Internalization of the programs’ goals to improve intrinsic motivation was facilitated by improving the community nutrition experts’ competence; the community nutrition experts are better at using their CommCare app after the intervention, which included components where community nutrition experts were trained on proper use of the app to support their work flow. Improvements in duration of counseling are also driven by improvements in reporting standards and increased knowledge in correct use of the app. Before the intervention, many community nutrition experts counseled their clients without opening the family counseling form leading to very low counseling times recorded on CommCare. After the intervention, and retraining on how best to use the family counseling form (ie, we encouraged them to open the form and fill in the details as they counsel), we see an increase in counseling time as a direct impact of our intervention. Accurate reporting is an essential component of performance in the CHW context due to the remote nature of their work and lack of immediate supervision, which means that any CHW’s activities, services provided, and changes in the client’s health status are not known without accurate data provided by the CHW. We also see improvements in reporting standards for form submissions.

Qualitative interviews with three community nutrition experts after the postintervention period support our interpretation that the calls improved the community nutrition experts’ competence and autonomy by improving knowledge and skills and helping to quickly solve any technical issues faced by the community nutrition expert. The continuous monitoring provided by the calls was seen to impact duration of counseling more than the other indicators, with the community nutrition expert giving more time to the client in addition to using the family counseling form while counseling to provide accurate reporting on this indicator.

Case activity also shows overall improvements for all community nutrition experts after our intervention, which is sustained in the postintervention period. Although we do not have a control group that did not receive any calls to provide concrete evidence, the results suggest that the calls, which included a strong component of supportive supervision, helped support autonomy and the internalization of program goals (which all community nutrition experts were aware of) leading to more autonomous motivation in the community nutrition experts and higher performance across all treatment groups. Our hypothesis that the calls lead to greater autonomous motivation is supported by the sustained improvements in duration of counseling in the postintervention period as a result of receiving feedback calls on duration of counseling and overall improvements in case activity, and improvements in reporting form submissions sustained after the intervention as well.

Providing CHWs with regular performance feedback through calls placed by a supervisor or manager is an effective way to reinforce goals and targets, and provide supportive supervision to CHWs encouraging their extrinsic and autonomous motivation and performance. Two-way communication opens avenues to discuss and provide solutions addressing gaps in performance, any challenges faced at work, or technical or systematic issues afflicting the CHW preventing her/him from performing effectively. Strategies targeting improvements in CHW programs should include an element of continuous monitoring and feedback system reinforced with supportive supervision to generate improvements in CHW performance and maintain an effective CHW program.

Other than the limitations posed by the dataset, our study has some important limitations that could potentially bias our outcomes:

1. Risks of unethical behavior leading to false reporting.

2. Spillovers where targets for different indicators are known to community nutrition experts in other groups.

3. Low network connectivity or unwillingness/inability of the community nutrition experts to respond to and answer the calls (could potentially be endogenous to performance if the community nutrition experts are aware of their own performance and do not answer the call if they perceive their performance to be low).

4. Holidays and events in week 3, which meant that the community nutrition expert(s) were absent with consent from their managers. Some may have extended their break. Attendance is a prerequisite to performance.

5. The most important limitation in the study arises because we do not have a control group that received no calls and no performance feedback. Hence, we cannot discern the effects of the calls themselves. This is important because the calls included other components other than the performance feedback, which can impact the community nutrition experts’ performance across all indicators, including the indicators on which they received no performance feedback.

In order to limit and test for the bias, we adopted the following strategies:

1. Draw an upper limit on the indicators where false reporting is most plausible. We imposed a limit of 20 minutes on the duration of counseling so that any outliers did not skew the mean counseling times.

2. Although we ensured that no community nutrition expert received feedback on other indicators other than the treatment she was assigned to, RMF reiterated the targets for all three indicators during the course of the study, which could have affected the community nutrition experts’ performance by setting goals. Goal setting can motivate performance, but we were interested in the effects of goal setting in conjunction with providing feedback.

3. We tested for correlation between initial performance and number of calls received to test whether community nutrition experts were deliberately avoiding calls anticipating unfavorable performance feedback. The pairwise correlation for baseline rank and number of calls received was *r*=–.265 (*P*<.001) suggesting that lower-performing community nutrition experts were less likely to answer calls.

## Appendix

Multimedia Appendix 1 Equations used to estimate main treatment effects and heterogeneous effects.

Multimedia Appendix 2 Results of matching based on ranks and treatment groups.

## Abbreviations

CHW: community health worker
DID: difference in difference
RMF: Real Medicine Foundation
SDT: self-determination theory
