# Supplementary material for: Effect of Performance Feedback on Community Health Workers’ Motivation and Performance in Madhya Pradesh, India: A Randomized Controlled Trial
Source: JMIR Public Health Surveill. 2016 Dec 7;2(2):e169. doi: 10.2196/publichealth.3381 (PMC5177738; doi:10.2196/publichealth.3381)
Supplement: Multimedia Appendix 1 [file publichealth_v2i2e169_app1.pdf]

We estimated main treatment effects and heterogeneous effects of call intensity (number of calls received) for each of the three performance indicators using a random effects model. We first estimated double difference estimates for main effects and heterogeneous effects in the intervention period, and then expanded the analysis to the post-postintervention period to estimate sustained effects of the intervention.

We estimated the following three equations using a random effects model to look for the main effects of the treatment for each indicator.

1. Main Effects: Double Difference Estimator

$$Performance_{ij} = \beta_0 + \beta_1 Treat_i + \beta_2 After_j + \beta_3 (Treat_i * After_j) + \epsilon_{ij} \quad (1)$$

We estimated three different specifications for this model with three different dependent variables for each of the performance indicators being assessed, namely case activity, form submissions number, and duration of counseling. For each of the specifications $Performance_{ij}$ is the performance of the community nutrition expert *i* in week *j*. $Treat_i$ is 1 if the community nutrition expert belongs to the treatment group and is 0 otherwise. $After_j$ is 1 if the data is from the intervention or post-intervention period, and 0 if it is from the baseline period. $Treat_i*After_j$ is 1 if Treat and After are both 1 and 0 otherwise. β_1 gives the difference in performance between the treatment and control groups in the baseline period (for difference in performance between the treatment and control in the intervention/post intervention period we can add β_1 and β_3), β_2 gives the difference between the baseline and intervention/post intervention period for the control group (for difference between the baseline and intervention periods for the treatment group we can add β_2 and β_3) , and β_3 gives us the double difference estimate, which is the estimated average effect of the treatment while accounting for baseline differences in performance and any change in performance overtime. ϵ_{ij} captures the error while β_0 gives us the constant. Our hypothesis is that β_3 will be significantly different than 0 for all three performance indicators.

We will estimate equation one using data only from the intervention period, weeks one to six, when the community nutrition experts were receiving calls, as well as using data from the intervention and post intervention periods, weeks one to ten to look for sustained effects of providing performance feedback on their performance.

2. Comparing each treatment against other two treatments as pooled controls: Cross Partial Effects

$$Performance_{ij} = \beta_0 + \beta_1 Treat_i + \beta_2 After_j + \beta_3 (Treat_i * After_j) + \epsilon_{ij} \quad (2)$$

After estimating the effect of performance feedback on the relevant indicator in equation 1, we estimate cross partial effects in equation 2, which is the effect of providing feedback on one indicator (x) on the performance in the other two indicators. That is to say we estimate the cross partial effects of providing feedback on case activity, on performance in duration of counseling, and form submissions, for each of the three indicators. The coefficients are to be interpreted similarly as in equation 1. Here too β_3 gives us the double difference estimates, which is our average treatment effect, the effect of providing performance feedback on one indicator on the performance in the other two indicators.

We run two different specifications for each of the three equations as in the first model

3. Comparing the Three Groups Head-to-Head

$$CaseActivity_{ij}=\beta_0+\beta_1 T_i^F+\beta_2 T_i^D+\beta_3(T_i^F * After_j)+\beta_4(T_i^D * After_j)+\beta_5 After_j+\epsilon_{ij} \quad (3)$$

$$FormSubmissions_{ij}=\beta_0+\beta_1 T_i^C+\beta_2 T_i^D+\beta_3(T_i^C * After_j)+\beta_4(T_i^D * After_j)+\beta_5 After_j+\epsilon_{ij} \quad (3)$$

$$Duration_{ij}=\beta_0+\beta_1 T_i^F+\beta_2 T_i^C+\beta_3(T_i^F * After_j)+\beta_4(T_i^C * After_j)+\beta_5 After_j+\epsilon_{ij} \quad (3)$$

Where T_i^F indicates that the community nutrition expert belongs to the Form Submissions treatment, T_i^C indicates that the community nutrition expert belongs to the Case Activity treatment, and T_i^D indicatates that the community nutrition expert belongs to the Duration of Counseling treatment.

In equation three we estimate the treatment effects of each treatment head to head against the other two treatments. The variables included in the equation are the same as in equation 1. The difference is that treatment dummies for two out of three treatments are included in the regression (in equation 1, we only include one treatment dummy, and treat the other two treatment groups as a pooled control group), and we omit the treatment group for the indicator being analyzed (i.e. if case activity is the response variable, then treatment group case activity is omitted from the regression) to compare the performance of the three treatment groups head to head on the different indicators. The three interaction terms are perfectly collinear, hence we drop the same indicator as the response variable from our model. We will repeat the estimation for each of the three indicators.

There are two null hypothesis: 1) β_1 = β_2 =0 , where we hope not to reject the null and find no baseline differences in the three treatment groups, and 2) $\beta_3=\beta_4$ =0, where we hope to reject the null and find that receiving feedback on a certain indicator e.g. Case Activity, affects performance on that indicator (case activity) differently than performance on the other two indicators (form submissions or duration of counseling). If the feedback is more effective in increasing performance on the other two indicators than on the feedback indicator, the coefficients for β_3 and β_4 will be negative.

4. Heterogeneous Effects

To test for heterogeneous effects of the treatment, i.e. call intensity, we interact the number of calls received with the treatment and period to obtain the effects of the number of calls received by the community nutrition expert in the treatment group. We estimate equation four.

$$Performance_{ij}=$$
$$\beta_0+\beta_1 Treat+\beta_2 After+\beta_3(Treat * After)+\beta_4 calls+\beta_5(calls * After)+\beta_6(calls * After * Treat)+E\,i$$
(4)

The coefficient of interest is β_6 which gives the heterogeneous impact of the treatment based on treatment intensity, i.e. Number of calls received in the intervention period. The sum of β_6 and β_3 give the double difference estimator measuring overall treatment effects where call intensity is also factored in. Here too, we run two different specifications, first limiting the data to the intervention period, and second including data from the post-intervention period to look for sustained effects of the treatment.
