# Supplementary material for: Effect of Performance Feedback on Community Health Workers’ Motivation and Performance in Madhya Pradesh, India: A Randomized Controlled Trial
Source: JMIR Public Health Surveill. 2016 Dec 7;2(2):e169. doi: 10.2196/publichealth.3381 (PMC5177738; doi:10.2196/publichealth.3381)
Supplement: Multimedia Appendix 2 [file publichealth_v2i2e169_app2.pdf]

Results of matching based on ranks and treatment groups

O=original, R=replaced; Marital Status: M=married; S=single; W=widow

	**Rank**		**Age**		**Edu**		**Start Year**		**Marital Status**		**Number of Children in Household**		**Number of Adults in Household**		**Participate in Other Work**	
**Treatment**	**O**	**R**	**O**	**R**	**O**	**R**	**O**	**R**	**O**	**R**	**O**	**R**	**O**	**R**	**O**	**R**
Form Submissions	19	16	32	24	8	12	‘11	‘10	M	M	2	2	6	5	0	1
Form Submissions	46	43	.	29	.	8	.	.	.	M	.	2	.	3	.	0
Form Submissions	49	53	31	26	12	16	‘12	.	M	M	4	0	2	6	0	0
Form Submissions	59	56	21	30	10	12	‘10	‘10	M	W	1	3	4	7	0	0
Case Activity	45	47	22	28	8	10	‘11	‘10	M	M	2	3	2	6	0	0
Case Activity	56	55	36	40	10	8	‘10	‘11	M	M	2	5	3	2	0	0
Duration of Counseling	42	44	23	28	12	16	.	‘11	M	S	1	0	3	5	0	0
Duration of Counseling	47	44	22	28	12	16	‘12	‘11	M	S	1	0	4	5	0	0
Duration of Counseling	51	53	31	28	12	12	‘10	‘10	M	S	3	0	4	5	0	1
Duration of Counseling	60	56	18	32	11	12	‘12	‘10	S	M	0	2	7	4	0	0
